# Supplementary material for: The Small RNA Universe of Capitella teleta
Source: Front Mol Biosci. 2022 Feb 25;9:802814. doi: 10.3389/fmolb.2022.802814 (PMC8915122; doi:10.3389/fmolb.2022.802814)
Supplement: Supplementary file 1 [file DataSheet1.ZIP › Supplement/known/CAPTEscaffold_181_12770.pdf]

Provisional ID : CAPTEscaffold_181_12770
Score total : 149834.7
Score for star read(s) : 3.9
Score for read counts : 149824.5
Score for mfe : 1.7
Score for randfold : 1.6
Score for cons. seed : 3
Total read count : 293886
Mature read count : 293472
Loop read count : 86
Star read count : 328

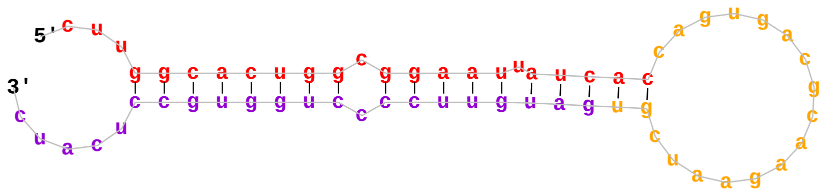

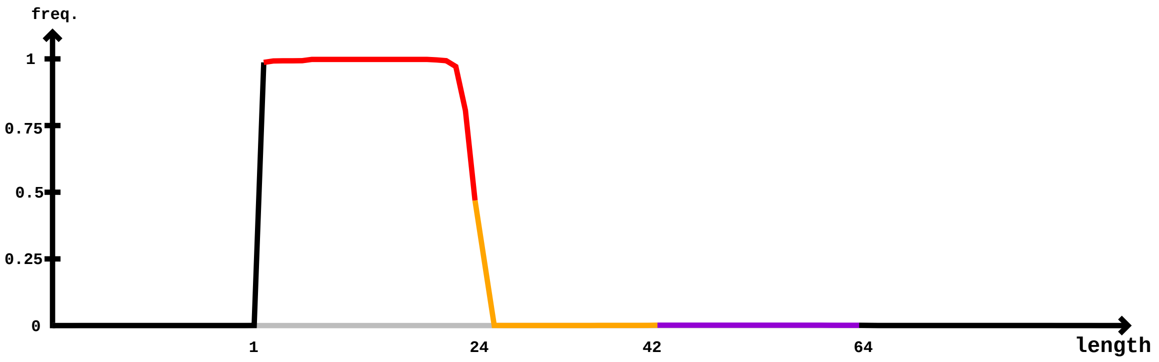

Mature Star

```
5'- uuguuuugcggaugguguguacuuggcacuggcggaauuaucaccagugacgcaagaaucgugauguuccccuggugccucaucucaccauccucccugucuuccuucauc  -3'  obs
    uuguuuugcggaugguguguacuuggcacuggcggaauuaucaccagugacgcaagaaucgugauguuccccuggugccucaucucaccauccucccugucuuccuucauc       exp
    uuguuuugcggaugguguguacuuggcacuggcggaauuaucaccagugacgcaagaaucgugauguuccccuggugccucaucucaccauccucccugucuuccuucauc       known
    .........((((((((.((...((((((((.(((((.(((((................))))))))))).))))))))...)).)))))))))..................   reads  mm  sample
    uuguuuugcggaugguguga..........................................................................................   8      0   seq
    uuguuuugcggauggugugG..........................................................................................   1      1   seq
    .uguuuugcggauggugugG..........................................................................................   8      1   seq
    .uguuuugcggaugguguga..........................................................................................   17     0   seq
    .uguuuugcggauggugugU..........................................................................................   1      1   seq
    ...................acuuggcacuggcggaauuauc.....................................................................   1      0   seq
    ...................acuuggcacuggcggaauuaucac...................................................................   42     0   seq
    ....................cAuggcacuggcggaauu........................................................................   1      1   seq
    ....................cuuggcacuggcggaaAu........................................................................   20     1   seq
    ....................cuuggcacuggcggaauA........................................................................   11     1   seq
    ....................cuuggcacuggcggaauu........................................................................   548    0   seq
    ....................cuuggcacuggcgCaauu........................................................................   1      1   seq
    ....................cuuggcacuggcAgaauu........................................................................   1      1   seq
    ....................cuGggcacuggcggaauu........................................................................   1      1   seq
    ....................cuuggcacuggcggaauua.......................................................................   823    0   seq
    ....................cuuggcacuAgcggaauua.......................................................................   1      1   seq
    ....................cuuggcacuUgcggaauua.......................................................................   1      1   seq
    ....................cuAggcacuggcggaauua.......................................................................   1      1   seq
    ....................cuuggcacuggcggaaCua.......................................................................   1      1   seq
    ....................cuuggcacuggcggaGuua.......................................................................   1      1   seq
    ....................cuuggcacuggcggaauuG.......................................................................   2      1   seq
    ....................cuGggcacuggcggaauua.......................................................................   1      1   seq
    ....................cuuggcacuAgcggaauuau......................................................................   5      1   seq
    ....................cuuggcacuggcggaauuaG......................................................................   2      1   seq
    ....................cuuggcacuggcggaUuuau......................................................................   4      1   seq
    ....................cuuggcacuggcggGauuau......................................................................   1      1   seq
    ....................cuuggcaUuggcggaauuau......................................................................   4      1   seq
    ....................cuuggcacugUcggaauuau......................................................................   2      1   seq
    ....................cuugAcacuggcggaauuau......................................................................   1      1   seq
    ....................cuuggcacuggcggaauuau......................................................................   6262   0   seq
    ....................Uuuggcacuggcggaauuau......................................................................   7      1   seq
    ....................cuuggcacuggcgUaauuau......................................................................   27     1   seq
```

```
                    Mature                                     Star
uuguuuugcggaugguguga cuuggcacuggcggaauuaucac cagugacgcaagaaucgu gauguuccccuggugccucauc ucaccauccucccugucuuccuucauc
....................cuuggUacuggcggaauuau......................................................................	3	1	seq
....................cuuggcacuggcCgaauuau......................................................................	3	1	seq
....................cuuggcGcuggcggaauuau......................................................................	1	1	seq
....................cuuggcacuggcggaaAuau......................................................................	1	1	seq
....................cuuggcacCggcggaauuau......................................................................	2	1	seq
....................cuuggcacuCgcggaauuau......................................................................	1	1	seq
....................Nuuggcacuggcggaauuau......................................................................	1	1	seq
....................cuuggcacuggcggaauAau......................................................................	2	1	seq
....................Auuggcacuggcggaauuau......................................................................	2	1	seq
....................cuuggcacuggcggaauuUu......................................................................	3	1	seq
....................cuuggcacuggcAgaauuau......................................................................	8	1	seq
....................cuuggcacuggcggaauuaA......................................................................	84	1	seq
....................cuuggcacGgcggaauuau......................................................................	1	1	seq
....................cAuggcacuggcggaauuau......................................................................	4	1	seq
....................cuuggcacuggcggaGuuau......................................................................	5	1	seq
....................cuuggcacuggcUgaauuau......................................................................	1	1	seq
....................cuuggcacuggAggaauuau......................................................................	2	1	seq
....................cuuggAacuggcggaauuau......................................................................	3	1	seq
....................cuuAgcacuggcggaauuau......................................................................	3	1	seq
....................cuuggcacugAcggaauuau......................................................................	1	1	seq
....................cuAggcacuggcggaauuau......................................................................	4	1	seq
....................cuuggcaGuggcggaauuau......................................................................	1	1	seq
....................cuuggcacAggcggaauuau......................................................................	3	1	seq
....................cuuggcacugCcggaauuauc....................................................................	3	1	seq
....................cuAggcacuggcggaauuauc....................................................................	36	1	seq
....................cGuggcacuggcggaauuauc....................................................................	2	1	seq
....................cuuggcacuggcggUauuauc....................................................................	5	1	seq
....................cuuggcacugUcggaauuauc....................................................................	2	1	seq
....................cuuggcacuggcggaUuuauc....................................................................	15	1	seq
....................cuuggcacCggcggaauuauc....................................................................	22	1	seq
....................cuuggcacuggcggaauuauG....................................................................	4	1	seq
....................cuuggcacuggcgUaauuauc....................................................................	2	1	seq
....................cuuggcacuggcggaaGuauc....................................................................	4	1	seq
....................cAuggcacuggcggaauuauc....................................................................	18	1	seq
....................cuuggcacuggcggaauuaCc....................................................................	7	1	seq
....................cuuggcacuggUggaauuauc....................................................................	11	1	seq
....................cuuggcGcuggcggaauuauc....................................................................	9	1	seq
....................cuuCgcacuggcggaauuauc....................................................................	1	1	seq
....................cuuggcacuggcggaauuaGc....................................................................	1	1	seq
....................cuuggcacuggcUgaauuauc....................................................................	5	1	seq
....................cuuggcacuggcggaauuGuc....................................................................	13	1	seq
....................cuuggcacuggcgAaauuauc....................................................................	11	1	seq
....................cuugAcacuggcggaauuauc....................................................................	16	1	seq
....................cuuggcUcuggcggaauuauc....................................................................	6	1	seq
....................cuuggcaUuggcggaauuauc....................................................................	12	1	seq
....................cuuggcacuggcggGauuauc....................................................................	7	1	seq
....................cuuggcacuggcggaauuauA....................................................................	55	1	seq
....................cuuggcacuAgcggaauuauc....................................................................	38	1	seq
....................cuuggcacuggcggaauuUuc....................................................................	3	1	seq
....................cuuggcaGuggcggaauuauc....................................................................	3	1	seq
....................cuuggcacuggcggaauuauU....................................................................	56	1	seq
....................cuuggGacuggcggaauuauc....................................................................	4	1	seq
....................cuuggcacuggcggaauCauc....................................................................	9	1	seq
....................cuuggcacuCgcggaauuauc....................................................................	14	1	seq
....................cuuggcaAuggcggaauuauc....................................................................	4	1	seq
....................cuuggcacuUgcggaauuauc....................................................................	3	1	seq
....................cuuggcacGgcggaauuauc....................................................................	3	1	seq
....................cuuggcacuggcggaauuaAc....................................................................	4	1	seq
....................cuuggcacuggcggaauuauc....................................................................	47438	0	seq
....................cuuggcacuggcggaaCuauc....................................................................	10	1	seq
....................cuuAgcacuggcggaauuauc....................................................................	24	1	seq
....................cuuggcacuggAggaauuauc....................................................................	19	1	seq
....................cuuggcacuggcggCauuauc....................................................................	1	1	seq
....................Auuggcacuggcggaauuauc....................................................................	7	1	seq
....................cuuggcacuggGggaauuauc....................................................................	7	1	seq
....................cCuggcacuggcggaauuauc....................................................................	4	1	seq
....................cuuggcCcuggcggaauuauc....................................................................	2	1	seq
....................cuuggcacuggcggaGuuauc....................................................................	6	1	seq
....................cNuggcacuggcggaauuauc....................................................................	3	1	seq
....................cuuggAacuggcggaauuauc....................................................................	23	1	seq
```

```
                    Mature                                   Star
uuguuuugcggauggugugacuuggcacuggcggaauuaucaccagugacgcaagaaucgugauguuccccuggugccucaucucaccauccucccugucuuccuucauc
....................cuugCcacuggcggaauuauc.....................................................................   2      1   seq
....................cuugUcacuggcggaauuauc.....................................................................   6      1   seq
....................cuuggcacuggcggaauuNuc.....................................................................   1      1   seq
....................cuCggcacuggcggaauuauc.....................................................................   6      1   seq
....................Nuuggcacuggcggaauuauc.....................................................................   20     1   seq
....................Guuggcacuggcggaauuauc.....................................................................   6      1   seq
....................cuuggcacAggcggaauuauc.....................................................................   7      1   seq
....................cuuggcacuggcgCaauuauc.....................................................................   2      1   seq
....................cuuggcacuggcAgaauuauc.....................................................................   62     1   seq
....................cuuggcacuggcggaauAauc.....................................................................   99     1   seq
....................cuuggcacugAcggaauuauc.....................................................................   2      1   seq
....................cuuggcacuggcggaauGauc.....................................................................   4      1   seq
....................cuuggcacuggcggaaAuauc.....................................................................   8      1   seq
....................cuuUgcacuggcggaauuauc.....................................................................   3      1   seq
....................cuuggcacuggcCgaauuauc.....................................................................   3      1   seq
....................cuuggcacuggcggaCuuauc.....................................................................   1      1   seq
....................cuuggUacuggcggaauuauc.....................................................................   26     1   seq
....................Uuuggcacuggcggaauuauc.....................................................................   47     1   seq
....................cuuggcacuggcCgaauuauca....................................................................   5      1   seq
....................cuuggAacuggcggaauuauca....................................................................   49     1   seq
....................cuuggcacuggcggaCuuauca....................................................................   2      1   seq
....................cuuggcacuggcggaauuauUa....................................................................   56     1   seq
....................cuGggcacuggcggaauuauca....................................................................   1      1   seq
....................cuugCcacuggcggaauuauca....................................................................   6      1   seq
....................cuuggGacuggcggaauuauca....................................................................   4      1   seq
....................cuugUcacuggcggaauuauca....................................................................   7      1   seq
....................cuuggNacuggcggaauuauca....................................................................   1      1   seq
....................cuuggcacAggcggaauuauca....................................................................   22     1   seq
....................cuuggcacuggcggaauuauAa....................................................................   70     1   seq
....................cuCggcacuggcggaauuauca....................................................................   20     1   seq
....................cuuggcacuggcggaauuauNa....................................................................   1      1   seq
....................cAuggcacuggcggaauuauca....................................................................   36     1   seq
....................cuuggcacuggcUgaauuauca....................................................................   20     1   seq
....................cuuggcacuggcggaauuauca....................................................................   97061  0   seq
....................cuuggcacuggcggaauuaCca....................................................................   9      1   seq
....................cuuggcUcuggcggaauuauca....................................................................   16     1   seq
....................cuuggcacuggcAgaauuauca....................................................................   135    1   seq
....................cGuggcacuggcggaauuauca....................................................................   2      1   seq
....................cuuggcacuggcggaGuuauca....................................................................   14     1   seq
....................cuuUgcacuggcggaauuauca....................................................................   13     1   seq
....................cuuggcacuggcgUaauuauca....................................................................   10     1   seq
....................cuuggcacCggcggaauuauca....................................................................   5      1   seq
....................cuuggcacugAcggaauuauca....................................................................   21     1   seq
....................cuuggcacuggcggGauuauca....................................................................   15     1   seq
....................cuuggcacuCgcggaauuauca....................................................................   15     1   seq
....................cCuggcacuggcggaauuauca....................................................................   5      1   seq
....................cuuCgcacuggcggaauuauca....................................................................   6      1   seq
....................cuuggcGcuggcggaauuauca....................................................................   25     1   seq
....................cuuggUacuggcggaauuauca....................................................................   29     1   seq
....................Guuggcacuggcggaauuauca....................................................................   4      1   seq
....................cuuggcacuggcggaaGuauca....................................................................   5      1   seq
....................cuuggcacuggcggaauGauca....................................................................   3      1   seq
....................cuAggcacuggcggaauuauca....................................................................   68     1   seq
....................cuuggcacuggcgCaauuauca....................................................................   2      1   seq
....................cuuggcacugUcggaauuauca....................................................................   7      1   seq
....................cuuggcacGggcggaauuauca....................................................................   8      1   seq
....................cuuggcacuggUggaauuauca....................................................................   19     1   seq
....................cuuggcacuggcggaauCauca....................................................................   13     1   seq
....................cuuggcacuggcggaauuUuca....................................................................   12     1   seq
....................cuuAgcacuggcggaauuauca....................................................................   50     1   seq
....................cuuggcCcuggcggaauuauca....................................................................   4      1   seq
....................cuuggcacuggcggaaCuauca....................................................................   10     1   seq
....................cuuggcacuUgcggaauuauca....................................................................   5      1   seq
....................cuuggcaUuggcggaauuauca....................................................................   10     1   seq
....................cuuggcacuggcggaUuuauca....................................................................   29     1   seq
....................Auuggcacuggcggaauuauca....................................................................   10     1   seq
....................cNuggcacuggcggaauuauca....................................................................   6      1   seq
....................cuuggcacuggcggaauuCuca....................................................................   1      1   seq
....................cuuggcacuggcggaauuaucG....................................................................   75     1   seq
....................cuuggcacuggcggaauAauca....................................................................   21     1   seq
```

Mature / Star			
uuguuuugcggaugguguga**cuuggcacuggcggaauuaucac**cagugacgcaagaaucgu**gauguucccuggugccucauc**ucaccauccucccugucuuccuucauc			
....................cuuggcaGuggcggaauuauca...	5	1	seq
....................cuuggcacuggcggaauuaGca...	7	1	seq
....................cuuggcacuggcggUauuauca...	18	1	seq
....................cuugAcacuggcggaauuauca...	93	1	seq
....................cuuggcacuggcggaauuauGa...	4	1	seq
....................cuuggcacuggcggaauuaAca...	24	1	seq
....................cuuggcacuAgcggaauuauca...	67	1	seq
....................cuuggcacuggcggaauuGuca...	16	1	seq
....................cuuggcacugCcggaauuauca...	4	1	seq
....................cuuggcacuggcgAaauuauca...	7	1	seq
....................cuuggcacuNgcggaauuauca...	1	1	seq
....................Nuuggcacuggcggaauuauca...	25	1	seq
....................cuuggcacuggAggaauuauca...	43	1	seq
....................cuuggcacuggcggaaAuauca...	27	1	seq
....................cuuggcaAuggcggaauuauca...	11	1	seq
....................cuuggcacuggcggaauuaucU...	365	1	seq
....................cuuggcacuggGggaauuauca...	15	1	seq
....................Uuuggcacuggcggaauuauca...	94	1	seq
....................cuuggcacuggcggaauuaucC...	3	1	seq
....................cuuggcacuggcggGauuaucac..	32	1	seq
....................cuuggUacuggcggaauuaucac..	53	1	seq
....................cuuggcaUuggcggaauuaucac..	8	1	seq
....................cuAggcacuggcggaauuaucac..	81	1	seq
....................cuugUcacuggcggaauuaucac..	17	1	seq
....................cuuggcacuggcggaauuaGcac..	4	1	seq
....................cuugCcacuggcggaauuaucac..	8	1	seq
....................cuuggcacuggcggUauuaucac..	43	1	seq
....................cuuggcacuggcggaauuaucUc..	28	1	seq
....................cuuggcacugCcggaauuaucac..	5	1	seq
....................Guuggcacuggcggaauuaucac..	9	1	seq
....................cuCggcacuggcggaauuaucac..	11	1	seq
....................cuuggcUcuggcggaauuaucac..	26	1	seq
....................Auuggcacuggcggaauuaucac..	20	1	seq
....................cuuggcacuggcggaauuaucaG..	2	1	seq
....................cuuggcacCggcggaauuaucac..	12	1	seq
....................cuuggcacuggcggCauuaucac..	2	1	seq
....................cuuggGacuggcggaauuaucac..	15	1	seq
....................cuuggcacuCgcggaauuaucac..	14	1	seq
....................cuuggcCcuggcggaauuaucac..	4	1	seq
....................cuuggcacuggcgNaauuaucac..	1	1	seq
....................cuuggcacuggcggaGuuaucac..	24	1	seq
....................cuuggcacuggcUgaauuaucac..	21	1	seq
....................cuuggcacuggGggaauuaucac..	10	1	seq
....................cuuggNacuggcggaauuaucac..	3	1	seq
....................cuGggcacuggcggaauuaucac..	2	1	seq
....................cuuggcacuggcggaaAuaucac..	22	1	seq
....................cuuggcacuggcggaauGaucac..	5	1	seq
....................cuuUgcacuggcggaauuaucac..	9	1	seq
....................cNuggcacuggcggaauuaucac..	5	1	seq
....................cuuggcacuggcggaauuaucac..	126242	0	seq
....................cuuggcacuggcggaCuuaucac..	1	1	seq
....................cuuggcacuggcggaUuuaucac..	12	1	seq
....................cuuggcacuggcggaauuaCcac..	14	1	seq
....................cuuggcGcuggcggaauuaucac..	25	1	seq
....................Uuuggcacuggcggaauuaucac..	85	1	seq
....................cuuggcacAggcggaauuaucac..	22	1	seq
....................cuuggcacuggcggaauuauAac..	33	1	seq
....................cuuggcacuggcggaauuUucac..	17	1	seq
....................cuuggcacuggcggaauuauNac..	1	1	seq
....................cuuggcacuggcggaauuaucaU..	727	1	seq
....................cuuggcacuggcggaauuaucaA..	834	1	seq
....................cCuggcacuggcggaauuaucac..	8	1	seq
....................cuuggcacuggcggaauuauUac..	46	1	seq
....................cuuggcacuggUggaauuaucac..	30	1	seq
....................cuuggcacuggcggaauuCucac..	1	1	seq
....................cuuggcacuggcgUaauuaucac..	14	1	seq
....................cuuggcacuggcggaauuauGac..	8	1	seq
....................cuuggcacuggAggaauuaucac..	35	1	seq
....................cuuAgcacuggcggaauuaucac..	68	1	seq
....................cuuggcacuggcggaaNuaucac..	1	1	seq

Mature   Star

```
uuguuuugcggaugguguga cuuggcacuggcggaauuaucac cagugacgcaagaaucgu gauguuccccuggugccucauc ucaccauccucccugucuuccuucauc
....................cuuggcNcuggcggaauuaucac...................................................................  1     1  seq
....................cuuggcacuggNggaauuaucac...................................................................  1     1  seq
....................cGuggcacuggcggaauuaucac...................................................................  2     1  seq
....................cAuggcacuggcggaauuaucac...................................................................  46    1  seq
....................cuuggcacuggcggaauCaucac...................................................................  22    1  seq
....................cuuggcacuggcggaauuaucaN...................................................................  2     1  seq
....................cuuggcaAuggcggaauuaucac...................................................................  12    1  seq
....................cuuggAacuggcggaauuaucac...................................................................  63    1  seq
....................cuuggcacuggcgAaauuaucac...................................................................  9     1  seq
....................cuuggcacugUcggaauuaucac...................................................................  4     1  seq
....................cuuggcacuggcggaaGuaucac...................................................................  4     1  seq
....................cuuggcacugAcggaauuaucac...................................................................  18    1  seq
....................cuuggcaGuggcggaauuaucac...................................................................  3     1  seq
....................Nuuggcacuggcggaauuaucac...................................................................  35    1  seq
....................cuuggcacGggcggaauuaucac...................................................................  13    1  seq
....................cuuggcacuggcggaauuGucac...................................................................  22    1  seq
....................cuuggcacuAgcggaauuaucac...................................................................  95    1  seq
....................cuuggcacuggcggaauAaucac...................................................................  114   1  seq
....................cuuggcacuggcggaauuaucGc...................................................................  29    1  seq
....................cuuggcacuggcgCaauuaucac...................................................................  8     1  seq
....................cuuggcacuggcggaauuaAcac...................................................................  43    1  seq
....................cuuggcacuggcAgaauuaucac...................................................................  161   1  seq
....................cuuCgcacuggcggaauuaucac...................................................................  12    1  seq
....................cuuggcacuggcCgaauuaucac...................................................................  1     1  seq
....................cuuggcacuggcggaaCuaucac...................................................................  11    1  seq
....................cuuggcacuUgcggaauuaucac...................................................................  14    1  seq
....................cuugAcacuggcggaauuaucac...................................................................  12    1  seq
....................Uuuggcacuggcggaauuaucacc..................................................................  1     1  seq
....................cuuggcacuggcggaaCuaucacc..................................................................  1     1  seq
....................cuuggcaAuggcggaauuaucacc..................................................................  1     1  seq
....................cuugAcacuggcggaauuaucacc..................................................................  1     1  seq
....................cuuAgcacuggcggaauuaucacc..................................................................  2     1  seq
....................cuuggcacuggcggaauCaucacc..................................................................  1     1  seq
....................cuuggcacuggcggaauuaucacA..................................................................  301   1  seq
....................cuuggcacuggcggaaGuaucacc..................................................................  1     1  seq
....................cuuggcacuggcggaauuGucacc..................................................................  1     1  seq
....................cuuggcacuggcggaauuaucacG..................................................................  3     1  seq
....................cuuggcacuCgcggaauuaucacc..................................................................  1     1  seq
....................cuuggcacuggcgAaauuaucacc..................................................................  1     1  seq
....................cuuggcacuggAggaauuaucacc..................................................................  1     1  seq
....................cuuggcacuggcggaaAuaucacc..................................................................  1     1  seq
....................cuuggcaGuggcggaauuaucacc..................................................................  1     1  seq
....................cuuggcUcuggcggaauuaucacc..................................................................  2     1  seq
....................cuugCcacuggcggaauuaucacc..................................................................  1     1  seq
....................cuAggcacuggcggaauuaucacc..................................................................  2     1  seq
....................cuuggcacuggcAgaauuaucacc..................................................................  3     1  seq
....................cuuggcacuAgcggaauuaucacc..................................................................  3     1  seq
....................cuuggcacuggcggaauuaucacc..................................................................  2054  0  seq
....................cAuggcacuggcggaauuaucacc..................................................................  1     1  seq
....................cuuggcacuggcggaauuaucacU..................................................................  3228  1  seq
....................cuuggcacuggcggaauuaucacAa.................................................................  10    1  seq
....................cuuggcacuggcggaauuaucaccC.................................................................  1     1  seq
....................cuuggcacuggcggaauuaucacca.................................................................  8     0  seq
....................cuuggcacuggcggaauuaucaccU.................................................................  9     1  seq
....................cuuggcacuggcggaauuaucaccaU................................................................  11    1  seq
.....................uuggcacuggcggaauuau......................................................................  4     0  seq
.....................uuggcacuggcggaauuauc.....................................................................  140   0  seq
.....................Cuggcacuggcggaauuauc.....................................................................  2     1  seq
.....................uuggcacuggcggaUuuauc.....................................................................  1     1  seq
.....................Auggcacuggcggaauuauca....................................................................  1     1  seq
.....................uuggcacuggcggaauuGuca....................................................................  1     1  seq
.....................Cuggcacuggcggaauuauca....................................................................  8     1  seq
.....................uuggcacuggcAgaauuauca....................................................................  1     1  seq
.....................uuggcacuggcggGauuauca....................................................................  1     1  seq
.....................uuggcacuggcggaauuauca....................................................................  318   0  seq
.....................uuggcacuggcggaauuaCca....................................................................  1     1  seq
.....................uuggcaAuggcggaauuauca....................................................................  1     1  seq
.....................uuggcacuggcggaauuaucaA...................................................................  3     1  seq
.....................uuggcacuggcAgaauuaucac...................................................................  2     1  seq
.....................uAggcacuggcggaauuaucac...................................................................  1     1  seq
```

```
                    Mature                                      Star
uuguuuugcggaugguguga cuuggcacuggcggaauuaucac cagugacgcaagaaucgu gauguuccccuggugccucauc ucaccauccucccugucuuccuucauc
uuguuuugcggaugguguga cuuggcacuggcggaauuaucaccagugacgcaagaaucgugauguuccccuggugccucaucucaccauccucccugucuuccuucauc
.....................uuggcacuggcggaauuaucac...................................................................	516	0	seq
.....................uuggcacuggcggaaAuaucac...................................................................	2	1	seq
.....................uuggcacuCgcggaauuaucac...................................................................	1	1	seq
.....................Cuggcacuggcggaauuaucac...................................................................	2	1	seq
.....................Auggcacuggcggaauuaucac...................................................................	1	1	seq
.....................uuggcacuggcggGauuaucac...................................................................	1	1	seq
.....................uuggcacGggcggaauuaucac...................................................................	1	1	seq
.....................uuggcacuAgcggaauuaucac...................................................................	1	1	seq
.....................uuggcacuggcggaauuaucaU...................................................................	3	1	seq
.....................uuAgcacuggcggaauuaucac...................................................................	1	1	seq
.....................uugAcacuggcggaauuaucacc..................................................................	1	1	seq
.....................uuggcacuAgcggaauuaucacc..................................................................	1	1	seq
.....................uAggcacuggcggaauuaucacc..................................................................	1	1	seq
.....................uuggcacuggcggaauuaucacc..................................................................	474	0	seq
.....................uuggUacuggcggaauuaucacc..................................................................	1	1	seq
.....................uuggcacuggcggaaAuaucacc..................................................................	1	1	seq
.....................uuggcacuggcggaauuaucacU..................................................................	87	1	seq
.....................uuggcacuggcUgaauuaucacc..................................................................	1	1	seq
.....................Auggcacuggcggaauuaucacc..................................................................	1	1	seq
.....................uuggcacuggcggaauuaucacA..................................................................	1	1	seq
.....................uuggcacuggcggaauuauAacc..................................................................	1	1	seq
.....................uuggcacuggcggUauuaucacc..................................................................	1	1	seq
.....................uuggcacuggcggaauuaucacca.................................................................	2	0	seq
......................uggcacuggcggaauuauc.....................................................................	1	0	seq
......................uggcacuggcggaauuauca....................................................................	1	0	seq
......................Cggcacuggcggaauuauca....................................................................	2	1	seq
......................Cggcacuggcggaauuaucac...................................................................	2	1	seq
......................uggcacuggcggaauuaucac...................................................................	51	0	seq
......................uAgcacuggcggaauuaucacc..................................................................	1	1	seq
......................uCgcacuggcggaauuaucacc..................................................................	1	1	seq
......................ugAcacuggcggaauuaucacc..................................................................	1	1	seq
......................uggcacuggcggaauuaucacU..................................................................	2	1	seq
......................uggcacuggcggaauuaucacc..................................................................	92	0	seq
......................uggcacuggcggaauuaucacca.................................................................	25	0	seq
........................gcacuggcggaauuauca....................................................................	112	0	seq
........................gcacuggcggaauuaucacU..................................................................	1	1	seq
.........................cacuggcggaauuaucac...................................................................	1366	0	seq
.........................cacCggcggaauuaucac...................................................................	1	1	seq
.........................cacuggcggaauuUucac...................................................................	1	1	seq
.........................cacuggcggUauuaucac...................................................................	1	1	seq
.........................cacAggcggaauuaucac...................................................................	1	1	seq
.........................cacuggcggaauuaucaU...................................................................	1	1	seq
.........................cacuggcAgaauuaucac...................................................................	3	1	seq
.........................cacuggcggGauuaucac...................................................................	1	1	seq
.........................cacuAgcggaauuaucac...................................................................	1	1	seq
.........................Uacuggcggaauuaucac...................................................................	3	1	seq
.........................cacuggcggaauuaucaA...................................................................	1	1	seq
.........................cacuggAggaauuaucac...................................................................	1	1	seq
.........................cacuggcggaauuaucacA..................................................................	2	1	seq
.........................cacuggcggaauuaucacU..................................................................	19	1	seq
.........................cacuggcggaauuaucacc..................................................................	23	0	seq
..........................................ccagugacgcaagaaucgu.................................................	1	0	seq
..........................................ccagugacgcaagaaucgug................................................	25	0	seq
...........................................cagugacgcaagaaucgu.................................................	59	0	seq
...........................................cagugacgcaagaaucgG.................................................	1	1	seq
....................................................aagaaucgugauguuccccuggugccu...............................	1	0	seq
.......................................................aaucgugauguuccccuAgugccu...............................	1	1	seq
.......................................................aauUgugauguuccccuggugccu...............................	1	1	seq
.......................................................aaucgugauguuccccuggugccu...............................	65	0	seq
.......................................................Uaucgugauguuccccuggugccu...............................	1	1	seq
.......................................................aaucgugauguuccccuggugccG...............................	11	1	seq
..........................................................cgugauguuccccuggugccuca.............................	1	0	seq
............................................................ugauguuccccuggugccu...............................	1	0	seq
............................................................ugauguuccccuggugccuc..............................	29	0	seq
............................................................ugauguuccccuggugccuca.............................	2	0	seq
............................................................ugauguuccccuggugccucau............................	3	0	seq
.............................................................gauguuccccuggugccuc..............................	4	0	seq
.............................................................gauguuccccuggugccuca.............................	1	0	seq
.............................................................gauguuccccuggugccucau............................	2	0	seq
.............................................................gauguuccccuggugccucauc...........................	256	0	seq
```

```
                    Mature                                  Star
uuguuuugcggaugguguga cuuggcacuggcggaauuaucac cagugacgcaagaaucgu gauguuccccuggugccucauc ucaccauccucccugucuuccuucauc
```

```
uuguuuugcggauggugugacuuggcacuggcggaauuaucaccagugacgcaagaaucgugauguuccccuggugccucaucucaccauccucccugucuuccuucauc
.............................................................gauguuccccuggCgccucauc...........................    1    1    seq
.............................................................Aauguuccccuggugccucauc...........................    2    1    seq
.............................................................gauguuAcccuggugccucauc...........................    1    1    seq
.............................................................gauguuUcccuggugccucauc...........................    1    1    seq
.............................................................gauguuccccuggugccucaucu..........................    1    0    seq
..............................................................auguuccccuggugccucauc...........................   23    0    seq
..............................................................auguuccccuggugccucaucu..........................    1    0    seq
...................................................................................ucaccauccucccugucuuc.......    1    0    seq
...................................................................................ucaccauccucccugucuucc......    1    0    seq
...................................................................................ucaccauccucccugucuuccu.....    1    0    seq
...................................................................................ucaccauccucccugucuuccuuc...    2    0    seq
...................................................................................ucaccauccucccugucuuccuuA...   38    1    seq
...................................................................................ucaccauccucccugucuuccuuAa..    1    1    seq
...................................................................................ucaccauccucccugucuuccuucaA.    1    1    seq
```
